# Junior-to-senior transition in men's sport under dual institutional constraints: a comparison of team and individual athletes

**DOI:** 10.3389/fspor.2026.1931153

**Published:** 2026-09-16

**Authors:** Rotem Marciano Mandil, Simcha Avugos, Sima Zach

**Affiliations:** School of Graduate Studies, Levinsky-Wingate Academic Center—Wingate Campus, Netanya, Israel

**Keywords:** athlete career development, junior-to-senior transition, sport type, talent development, youth sport

## Abstract

**Background:**

The transition from junior to senior sport is a multifaceted process involving psychological, physical, social, and organizational demands, and is conceptualized through several models of athletic development and career transitions. This study examined how Israeli junior athletes experience this transition, with particular attention to structural differences between individual (swimming) and team (basketball) sports and their implications for progression within elite sport pathways.

**Methods:**

A qualitative design combining deductive theory-informed analysis with inductive thematic analysis of field-based data was employed. Semi-structured interviews were conducted with 19 male athletes, aged 14–20, who were concurrently competing in youth leagues and training at senior level.

**Results:**

Five interrelated domains emerged: entry pathways, changing performance demands, integration and support, military service as structural constraint, and retrospective evaluation. Swimming transitions were institutionally structured and selection-based, emphasizing technical refinement and residential cohesion. Basketball transitions were market-driven and relationally negotiated, characterized by intensified physical demands, performance pressure, and hierarchical integration. Military service functioned as a systemic bottleneck for swimmers and a logistical strain for basketball players.

**Conclusion:**

The findings highlight how sport typology and cultural context critically shape transition experiences, extending understanding of junior-to-senior transitions as embedded within interacting sport-specific structures and broader sociocultural conditions.

## 1 Introduction

Athletes typically progress through three development stages: sampling (ages 6–12), specializing (13–15), and investment (16+), although age ranges vary across sports (1). The transition to senior-level competition generally occurs during the investment stage and is marked by intensified commitment, higher performance standards, and increased professional expectations (2, 3). From a high-performance sport perspective, this stage represents the critical entry point into sustained elite-level performance environments. While it offers opportunities for advancement, it also exposes athletes to substantial physical, mental, and social demands (4). Whether athletes consolidate or discontinue elite participation is often determined during this transition.

Psychological readiness is central to this process. Mental resilience, self-efficacy, emotional regulation, and effective goal-setting help athletes manage the pressure associated with senior competition, including intensified training, performance scrutiny, and reduced tolerance for mistakes (5–7). Insufficient psychological preparation increases vulnerability to stress, burnout, and dropout (8). Accordingly, structured psychological support through sports psychologists, mentors, or targeted coping interventions can enhance athletes’ capacity to adjust and persist. These factors are increasingly recognized as determinants of performance continuity at the elite level.

Social support systems are equally influential. Coaches, family members, and peers provide emotional reassurance, practical guidance, and performance-related feedback during periods of uncertainty (3, 9). Coaches are particularly influential in shaping motivational climates, setting expectations, and fostering belonging, while peers can reduce isolation and ease integration into senior environments (10). However, these dynamics may differ across sport types: team-sport athletes often rely heavily on peer relationships, whereas individual-sport athletes tend to depend more on coaches and family (8). Understanding these contextual differences is essential for tailoring effective transition support. Such tailoring is particularly relevant in high-performance systems where marginal gains in social integration can influence selection and retention at senior level.

The physical dimension of transition is also substantial. Senior-level competition requires athletes to refine technical and tactical skills, increase strength and endurance, and adapt to greater speed, intensity, and physicality (11). Structured training programs that progressively increase load and complexity are critical for bridging the gap between junior and senior standards [e.g., (12–15)]. Beyond training volume, qualitative shifts in precision, recovery practices, and professional discipline often distinguish successful transitions.

Organizational environments further shape transition experiences. Clear developmental pathways, access to training resources, and competitive opportunities reduce uncertainty and facilitate progression (16). Dual-career programs that integrate education or vocational training alongside sport can buffer stress and promote long-term career stability (17). In contrast, unclear progression routes or limited institutional support may intensify transition-related strain. Such organizational variability has direct implications for talent retention and performance sustainability in elite pathways.

Cultural context adds another layer of complexity. Societal values, national sport systems, and educational expectations influence how athletes prioritize and experience sport participation (18, 19). In some contexts, commercialization pressures young athletes to compete for scholarships or professional contracts (20). Personality traits and individual differences further shape adaptation trajectories (21). Drew et al. (22) propose a comprehensive framework integrating individual, environmental, and cultural factors, highlighting the multidimensional nature of junior-to-senior transition. These contextual influences are increasingly acknowledged as shaping elite performance trajectories across national sport systems.

Several theoretical models provide structured lenses for understanding this process. Stambulova's Athletic Career Transition Model (5) conceptualizes transitions as a balance between resources (e.g., coping skills, social support) and barriers (e.g., injury, insufficient guidance). Successful adaptation occurs when resources outweigh demands. Wylleman and Lavallee's Holistic Athletic Career Model (23) expands this perspective by conceptualizing transitions across athletic, psychological, academic, and social domains, highlighting dual-career demands and the key role of coaches and family in supporting life balance. Côté's Developmental Model of Sport Participation (3) emphasizes long-term skill development and early diversification as foundations for later elite performance. Henriksen et al.'s Ecological Model (16) situates athlete development within broader environmental systems, while Danish et al.'s Life Development Intervention Model (24) highlights transferable life skills such as goal-setting and time management. Taylor and Ogilvie's model (25) focuses on identity-related challenges in career transition. Together, these frameworks emphasize that transition outcomes reflect interactions between personal capacities and environmental conditions rather than isolated factors.

Empirical research on junior-to-senior transitions has relied primarily on cross-sectional designs [e.g., (8, 26)] and, to a lesser extent, longitudinal studies [e.g., (21, 27)]. Studies span both individual sports (e.g., table tennis, tennis, swimming, horse riding) and team sports (e.g., soccer, basketball, rugby, hockey), using questionnaires [e.g., Transition Monitoring Survey; (28)] and qualitative interviews with athletes and coaches [e.g., (8, 18, 29)]. Broadly similar psychological demands have been reported across sport types, including identity recognition and performance-related pressure. Factors associated with successful transition include psychological adjustment and social support. Differences by sport type have been reported mainly in sources and forms of support, with team-sport athletes relying more on peer- and coach-based relationships within a group structure, whereas individual-sport athletes tend to depend more on personalized coach-athlete relationships. Despite this growing body of work, direct comparisons between individual and team sports within the same cultural context remain limited. This gap is particularly relevant for understanding how different sport systems produce distinct elite performance pathways.

This study examines the experiences of Israeli junior basketball players and swimmers transitioning to senior levels and contributes to the literature in several important ways. First, it compares transition experiences between team (basketball) and individual (swimming) sports, illuminating differences in support needs, coping strategies, and transition-related challenges—an area rarely explored comparatively. Because the timing of the junior-to-senior transition differs across sports, these comparisons are intended to identify patterns within the two sport contexts rather than isolate sport-type effects from developmental-stage differences. Second, it introduces a cultural dimension by focusing on Israeli athletes, showing how national sport structures, family dynamics, and broader social expectations (e.g., cultural attitudes toward sports) shape the transition process in a non-European and American context. Third, it analyzes the role of key support systems—family, coaches, mentors, and sport organizations—and how their functions differ across team and individual settings. Fourth, it extends existing theoretical models by examining the interaction between psychological and environmental factors during this developmental stage. This integration provides a multi-dimensional perspective on the transition process. Finally, the study identifies coping strategies employed by junior athletes and links them to transition outcomes, offering practical implications for designing sport-specific support programs.

The Israeli context provides a distinctive backdrop for examining these transitions. According to the Israel Basketball Association (https://ibasketball.co.il), only a small proportion of youth players (≈11%) reach senior Premier or National League rosters, indicating the selectivity of progression. Comparable data in other sports are limited. In addition, young Israeli athletes must navigate high-performance sport with academic demands, often within limited formalized dual-career structures. Mandatory military service at age 18 introduces a further structural constraint that may disrupt athletic development. Unlike some systems in which elite athletes may receive full exemption from military service (e.g., South Korea; 30), Israel's arrangements allow elite athletes to continue training through modified military service while remaining subject to the service obligation. Thus, the combined demands of sport, education, and military service create a uniquely complex transition environment. This makes the Israeli case particularly informative for understanding elite athlete development under multiple institutional constraints.

Given the exploratory nature of the study and limited comparative research on junior-to-senior transitions across sport types, a qualitative, comparative, cross-sectional design was appropriate for capturing athletes’ retrospective accounts of their experiences, challenges, and sources of support, while considering the broader structural and cultural context of the Israeli sporting environment. This design provided in-depth access to athletes’ perspectives in two relatively small elite sport systems, while recognizing that it does not permit direct observation of change over time or fully disentangle sport type from related factors such as age.

## 2 Methods

### 2.1 Participants

The participants were 19 young male athletes, aged 14–20, including 10 basketball players (aged ≥ 18, M_age_ = 18.9, SD = 0.73) and 9 swimmers (M_age_ = 16.6, SD = 1.73). The age difference between the groups reflects differences in the typical timing of the junior-to-senior transition in the two sports in Israel: basketball players generally enter senior-level competition at age 18 or later, whereas swimmers may begin competing at the senior level at younger ages. Accordingly, eligibility was based on athletes’ transition status and senior-level experience rather than chronological age alone.

Basketball and swimming were purposively selected to represent contrasting team and individual sport contexts, respectively, as both attract substantial youth participation. Both sports have national federations, structured age-category competition, and defined pathways into senior-level competition in Israel. However, basketball senior opportunities can involve a player-agent/recruitment market and professional club leagues, whereas swimming has no comparable domestic professional structure; advancement to senior-level competition is primarily mediated through federation- and coach-led selection into elite training programs. For swimmers, the closest equivalent to “turning professional” is selection into structured national or Olympic training groups.

All participants were actively competing as juniors while concurrently training at the senior level. Consistent with Drew et al. (22), eligibility required between one month and one year of senior-level training to capture the transition phase. Basketball players transitioned to either the Premier or National League. Participants were recruited through club coaches and were required to demonstrate sufficient language proficiency to participate in the study. All provided written informed consent (with parental consent for minors). Confidentiality and anonymity were assured, and no compensation was provided.

### 2.2 Research design and tools

A cross-sectional design was employed to examine athletes’ perspectives on their transition from junior to senior sport. Consistent with established qualitative approaches in the field [e.g., (4, 21, 22, 31, 32)], data were collected through in-depth, semi-structured interviews to capture lived experiences. Interviews explored transition experiences, coping strategies, support systems, and the impact of mandatory military service.

### 2.3 Procedure

The study received approval from the Institutional Review Board (IRB-00426). Face-to-face interviews were conducted in private settings and lasted 30–60 min. All interviews were audio-recorded using a mobile device.

### 2.4 Data analysis

The study employed a hybrid analytic approach, integrating theory-driven (deductive) coding with inductive thematic analysis based on field data (33, 34). Interviews were transcribed verbatim and analyzed to identify recurring themes (35). Themes were grouped into categories and organized by sport type (team vs. individual). Following Drew et al. (22), we first identified personal, external, and cultural themes, which were further refined into subcategories. In a second analytic phase, additional themes specific to the Israeli context were identified [cf. (36, 37)]. Coding was conducted jointly by two authors [(RM; SZ)], who discussed and reached consensus on the coding of each transcript. The third author [(SA)] reviewed the coding scheme and resolved any remaining ambiguities. Because coding was consensus-based rather than independent, an inter-rater reliability statistic (e.g., Cohen's Kappa) was not computed. An audit trail documenting coding decisions was maintained to support dependability (see Section 2.5).

### 2.5 Trustworthiness and rigor

Trustworthiness was ensured by addressing credibility, transferability, dependability, and confirmability [e.g., (38, 39)]. Credibility was supported through sustained engagement with participants and investigator cross-checking of the findings. Transferability was strengthened by providing rich, detailed descriptions of the participants and context, allowing readers to assess the applicability of the findings to other settings. Finally, dependability and confirmability were maintained through an audit trail, documenting all decisions and changes made during the research process to ensure transparency.

## 3 Results

The thematic analysis of the interviews generated five interrelated domains shaping the junior-to-senior transition: (1) Entry pathways to senior sport, (2) Changing performance demands and professional expectations, (3) Support, coping, and integration, (4) Military enlistment as a cultural-structural constraint, and (5) Retrospective evaluation and future orientation.

Within each domain, a comparative analysis of swimmers and basketball players revealed patterned differences between a structured, institutionally mediated pathway (swimming) and a market-driven, relationally negotiated transition pathway (basketball), alongside several shared adjustment challenges.

Table 1 summarizes the five thematic domains, their sport-specific subthemes, and an illustrative quotation for each. As the design was cross-sectional, all descriptions below reflect athletes’ retrospective accounts of their transition rather than change tracked prospectively over time.

**Table 1:** Junior-to-senior transition: thematic domains, subthemes, and illustrative quotations by sport.

Domain	Sport	Subtheme	Illustrative quote
Entry pathways	Swimming	Institutional selection (Academy admission based on performance standards)	"There was a very good competition… The [head] coach approached my coach and my parents and offered me this option.” (I.R.)
	Basketball	Market-driven negotiation via personal agents	"An offer was conveyed through my agent.… I decided it was the right opportunity to pursue.” (I.D.)
Performance demands	Swimming	Technical/professional intensification	"The dry, meticulous warm-up and stretching after workout… here they're meticulous, and I love it.” (A.G.)
	Basketball	Physical shock and reduced tolerance for error	"In the seniors, you have far fewer mistakes you can afford to make.” (I.D.)
Integration and support	Swimming	Residential cohesion, pre-existing peer networks	"A few athletes had been abroad with me..Yes, I already knew all of them.” (E.F.)
	Basketball	Hierarchical, status-sensitive integration	”..in seniors no one really pays attention to you, and sometimes you barely play.” (O.C.)
Military service	Swimming	Career bottleneck (dependent on “active/outstanding athlete” status)	"It's unfortunate that many swimmers drop out at 18 because there are not enough “active athlete” status positions.” (O.V.)
	Basketball	Dual-commitment logistical burden	"It just slightly affects your peace of mind because you have another framework, unlike other teammates.” (Z.V.)
Retrospective evaluation	Swimming	Holistic, dual-career-oriented growth	"Even if something felt bad at the time, at the end, it all helped me improve.” (I.R.)
	Basketball	Competitive advancement, upward mobility	"Those struggles built me up and made me stronger than I was before.” (L.B.)

### 3.1 Entry pathways: institutional selection vs. market negotiation

#### 3.1.1 Swimming

For swimmers, the transition to the senior level was primarily organizational rather than contractual. Two distinct pathways emerged: entry into the Wingate Academy of Excellence and continued progression within local clubs. Admission to the Academy was described as a selective, performance-based process following national-level success. Recruitment signaled formal recognition of elite potential and marked a decisive career turning point. For the majority of participants, it required relocation and separation from home. One swimmer described how the head coach approached him after a strong competition:

“There was a very good competition, with very good results…. the [head] coach approached my coach and my parents and offered me this option.” (I.R.)

Athletes framed the move to the Academy as a step toward high-performance sport specialization, emphasizing access to superior facilities, structured daily routines, and training alongside an elite peer group. In contrast, swimmers who remained in their clubs described a more gradual, age-based progression (typically around 14–15), characterized by continuity rather than major environmental change.

Emotionally, Academy swimmers reported both excitement and heightened expectations, whereas club swimmers mainly anticipated an increase in training demands rather than a fundamental shift in their sporting environment.

#### 3.1.2 Basketball

In contrast to the structured swimming pathway observed above, the transition in basketball was largely transactional and market-driven. Half of the participants relied on personal agents to navigate entry into the senior league. Four players remained with the senior team of the same club in which they had competed at the youth level, while others pursued external opportunities. As one player explained:

“An offer was conveyed through my agent. He informed me that Netanya was interested, and after considering my options, I decided it was the right opportunity to pursue.” (I.D.)

The primary consideration in selecting a team was anticipated playing time. Players and their agents evaluated potential clubs not on prestige alone, but on the likelihood of securing meaningful minutes on the court:

“To play—mainly to play—not to sit on the bench.” (R.B.)

At the same time, several participants emphasized the importance of developmental support, including a coaching staff committed to nurturing young talent and the presence of similarly aged teammates:

“First of all, a place that really believes in developing young players, and a support system that includes a personal trainer.” (N.LM.)

This strategic positioning process—carefully choosing a team to maximize development and exposure—generated both excitement and concerns. While players expressed enthusiasm about entering the professional arena, they also described the pressure associated with meeting first-division expectations at a young age. As young players, they felt that their entire career trajectory depended on finding a coach willing to trust a “rookie":

“I was very enthusiastic and excited about it. And that's a good thing—but it's also difficult to meet the expectations of the first division as a 16–17-year-old.” (L.B.)

Comparison: Swimming transitions were institutionally structured and selection-based, whereas basketball transitions were individually negotiated within a competitive market logic centered on playing opportunities.

### 3.2 Shifting performance demands: precision vs. physicality

#### 3.2.1 Swimming

Swimmers did not perceive their transition as involving a dramatic increase in water training volume (typically 8–9 sessions per week); rather, they experienced it retrospectively as a qualitative intensification. They described a shift toward what they termed “independent professionalism”: greater attention to technical precision, structured dry-land training, recovery routines, and greater personal responsibility:

“Here I have gym training twice and mobility once. Before, I had nothing. I would arrive five minutes before practice and go straight into the water.” (E.F.)

“There are things I wouldn't have noticed professionally such as the dry, meticulous warm-up and stretching after workout, which I used to do more haphazardly. Here they're meticulous, and I love it.” (A.G.)

“There is a strong emphasis on maintaining technique. We swim long distances with a focus on precision. In clubs, this level of professionalism is less present.” (A.F.)

The Academy environment provided multidisciplinary support, including access to nutritionists and regular psychological counseling. Psychological services were integrated as part of elite preparation rather than as remedial intervention—supporting competition readiness, cognitive load, and emotional regulation. As one swimmer noted:

“Through the Academy, I have a psychologist. I speak with her about twice a month.. about everything—preparation for training, preparation for important or less important competitions, and situations that arise in specific training sessions.” (I.R.)

In contrast, club swimmers did not report a substantial structural transformation in their training environment, nor did they express dissatisfaction with professional guidance. Rather, they described an incremental increase in workload—typically one additional water session per week and expanded fitness training—without broader systemic changes in support provision.

#### 3.2.2 Basketball

Players commonly experienced the transition to senior basketball as a “physical shock,” involving a sharp increase in game intensity, defensive aggression, and tactical speed. One player noted:

“In youth I could be among the highest, there [in seniors] I could be average… much more aggressive.” (R.B.)

On the mental level, a recurring theme was the “burden of proof” in senior competition. Players emphasized that mistakes were no longer treated as part of the learning process but as performance liabilities:

“In the seniors, you have far fewer mistakes you can afford to make. You have to do everything at 100% and mistakes aren't allowed.” (I.D.)

In addition, participants described a broader shift in the sporting environment from developmental to business-oriented. This included heightened expectations regarding responsibility, availability, and performance consistency:

“The pressure at the end of every game.. it can involve management, money, and sponsors.” (R.B.)

Comparison: Swimming emphasized technical refinement and structured professionalization of training, whereas basketball emphasized intensified competition, physicality, and reduced tolerance for error.

### 3.3 integration and support: cohesion vs. hierarchy

#### 3.3.1 Swimming

Despite increased demands—which typically required one or two months of adjustment—social integration was generally described as smooth. Several Academy swimmers had previously competed together on youth national teams, which reduced feelings of social discontinuity. The residential environment fostered strong peer cohesion and helped mitigate the sense of isolation often associated with individual sports. As one swimmer explained:

“A few athletes had been abroad with me; others I met in national team camps and different training camps. Yes, I already knew all of them.” (E.F.)

At the same time, relationships became more performance-oriented, replacing earlier paternalistic dynamics. Emotional closeness was partially replaced by results-based expectations. Family remained the central emotional anchor. As one swimmer noted:

“My parents—both mom and dad—helped me a lot. They were there when I needed them, reassured and encouraged me.” (A.F.)

Coping was characterized by adaptive self-regulation strategies, including patience, self-discipline, and long-term goal orientation. The perceived need for formal psychological intervention varied individually.

#### 3.3.2 Basketball

Most players reported relatively smooth social integration, often due to peer similarity or shared youth-team progression:

“I integrated best socially. This is likely because nearly everyone on this team is young and close to my age.” (O.C.)

However, adaptation to senior performance expectations was gradual and sometime fragile. While some athletes adjusted within weeks, others required several months to meet senior performance standards. This adaptation process was sensitive to disruption, particularly following mid-season injuries or coaching changes:

“It took me some time to understand what was expected of me and what I was supposed to bring to the court.” (D.S.)

The shift from being a central player in youth leagues to a marginal or bench player at the senior level was frequently associated with a perceived status loss and reduced instructional attention from head coaches:

“In youth you don't stop playing and you are a key player, but in seniors no one really pays attention to you, and sometimes you barely play.” (O.C.)

Institutional psychological support was limited, leading some athletes to seek privet services:

“I personally go to someone [psychologist] I worked with back in the youth division.” (O.C.)

Support from coaching staff was typically delivered by assistant or professional staff members rather than the head coach. Family support remained central:

“Mainly family support, which is always there. My older brother helps me a lot.” (A.M.)

Finally, the presence of foreign players introduced linguistic and generational gaps that often limited relationships to professional interactions. Communication was frequently confined to on-court matters:

“I was less close to him since he was also older. Plus, it's different—he doesn't speak Hebrew, and he has his own habits.” (N.L.)

Comparison: Swimming integration relied on pre-existing networks and residential cohesion, whereas basketball integration was more hierarchical, status-sensitive, and dependent on informal or private support systems.

### 3.4 Military service as structural constraint and dual-commitment

#### 3.4.1 Swimming

Military enlistment emerged as a key structural and cultural constraint. Swimmers approaching age 18 perceived army service as potentially career-disruptive unless they secured official “active athlete” or “outstanding athlete” status. As one swimmer explained:

“My goal is to enlist as an “active athlete”—ideally as an “outstanding athlete”. To be accepted, you must reach a required performance standard.” (L.S.)

Athletes described this designation as essential for continuing their swimming careers into adulthood. Without it, sustained elite training would be nearly impossible. Military service therefore functioned as a structural bottleneck interacting with a narrow high-performance developmental window, intensifying transition pressures:

“It's unfortunate that many swimmers drop out at 18 because there are not enough “active athlete” status positions in the army.” (O.V.)

For those who secured athlete status, the challenge shifted toward maintaining high-intensity training while continuing to meet performance criteria.

#### 3.4.2 Basketball

For basketball players, securing “athlete” status required proactive planning during high school and close coordination with club management. As one participant noted:

“Honestly, there was no hesitation. I already knew a year before that this was my goal, so there wasn't much doubt.” (I.D.)

Another player added:

“Both the professional staff and management, as well as myself, aimed for me to receive “athlete” status. I was in the process with the army throughout the entire year.” (D.S.)

Nine players served under “active athlete” status and one as “outstanding athlete”, requiring continuous coordination between sport and military systems. Most participants described the dual commitment as physically demanding and mentally taxing, as it added an additional institutional framework to their already intensive schedules:

“It just slightly affects your peace of mind because you have another framework, unlike other teammates [who aren't soldiers].” (Z.V.)

Comparison: In both sports, military service functioned as a structural constraint. However, in swimming, it was framed primarily as a career bottleneck, whereas in basketball it was experienced as a dual-commitment logistical burden.

### 3.5 Retrospective evaluation and future aspirations

#### 3.5.1 Swimming

Most swimmers expressed overall satisfaction with their transition, describing it as maturing and character-building. They interpreted challenges—such as relocating from home and adapting to the intensity of Academy life—as essential for both athletic and personal growth. As one swimmer reflected:

“Even if something felt bad at the time, at the end, it all helped me improve.” (I.R.)

Participants emphasized the importance of structured mental and physical preparation to cope with increasing training loads and rising performance standards. They also highlighted the need to prepare young athletes for the non-linear nature of progression at the senior level:

“A swimmer who is the best on his team can suddenly be the last in the new team. It's not an easy transition, and we have to overcome it.” (E.M.)

Regarding coaching practices, swimmers stressed the importance of balancing high expectations with consistent communication and emotional support:

“I would advise the coach to push the athletes, but at the same time care for them and not exhaust them. He should be very supportive and understanding.” (A.G.)

Future aspirations included Olympic participation, European Championships, and NCAA pathways.

#### 3.5.2 Basketball

Players similarly framed the transition as developmental, emphasizing that the challenges they faced fostered both personal and professional growth. They valued not only their successes but also their setbacks as formative experiences. As one participant noted:

“I made mistakes every now and then, but I'm happy with that because I have learned important lessons.” (I.D.)

Another player added:

“Those struggles built me up and made me stronger than I was before. Moving up to the seniors also made me more mature personally, from a kid to a young man.” (L.B.)

In retrospect, they recommended that youth coaches place greater emphasis on defensive fundamentals and physical preparation in order to narrow the gap between youth and senior competition:

“I would recommend emphasizing defense as much as possible. Don't let it slide—address every detail, practice it, and work on it as much as needed. Many players transitioning from youth to seniors often get stuck or don't get minutes because of their defense.” (O.C.)

Aspirations focused on elite professional leagues and national representation. Beyond competitive advancement, they emphasized personal fulfillment and representing Israel at the senior national level:

“I wish to keep enjoying basketball my whole career…. and to truly reach the levels I aspire to—like the senior Israeli national team and the top teams in the country.” (A.M.)

Comparison: Both groups retrospectively framed the transition in developmental terms, including personal growth and maturity. Swimmers emphasized holistic preparation and dual-career sustainability, whereas basketball players emphasized competitive advancement and upward mobility within professional hierarchies.

## 4 Discussion

### 4.1 General discussion

This study examined the junior-to-senior transition through a comparative lens, revealing how entry structures, performance demands, integration dynamics, and dual institutional commitments interact to shape athletes' developmental trajectories. Grounded in established transition frameworks (5, 16, 23), the findings extend existing models by demonstrating that transitions are not only psychologically demanding but also structurally differentiated according to sport-specific organizational systems, with important implications for athlete development within high-performance sport environments.

A central contribution of this study lies in identifying two contrasting transition architectures. Swimming operated through institutional selection and centralized professionalization, particularly via the Academy framework, whereas basketball transitions were negotiated within a competitive market logic centered on playing opportunities and coach trust. This structural contrast suggests that transition experiences are shaped less by uniform emotional processes and more by differing levels of structural predictability. In swimming, selection into elite frameworks provided clarity and long-term direction, while also introducing heightened expectations. In basketball, athletes navigated contractual mobility, role instability, and performance visibility within a hierarchical team environment. This distinction refines Stambulova's (5) model by illustrating how the balance between demands and resources is embedded in organizational design, not merely individual appraisal.

These structural differences were also reflected in athletes' subjective experiences. Across both sports, participants reported identity-related challenges during the transition, consistent with prior research (4, 8). However, the nature of this disruption varied by sport context. Basketball players, particularly those entering the Premier League, described a loss of status associated with reduced playing roles and diminished visibility within senior squads. Swimmers, in contrast, experienced identity strain primarily in relation to performance benchmarks, especially when failing to meet qualifying standards, thereby linking self-evaluation more directly to measurable outcomes. These patterns reflect broader sport-type differences: disrupted social hierarchies in team sport versus internally driven performance pressures in individual sport. Collectively, they reinforce the need to evaluate transition experiences according to sport typology, as suggested by Côté (3) and Drew et al. (22).

Athletes' retrospective accounts suggest that the transition was experienced less as increased workload and more as a qualitative recalibration of performance demands. Swimmers described a shift toward technical precision, multidisciplinary support, and self-managed professionalism, whereas basketball players emphasized exposure to intensified physicality, reduced tolerance for error, and heightened performance accountability. These patterns suggest that transitions involve a redefinition of what it means to be “successful” within each sport context. For swimmers, advancement required refinement and procedural discipline. For basketball players, it required competitive resilience within performance hierarchies. This further indicates that performance demands are not uniform across sport typologies but are instead embedded within the cultural and organizational characteristics of each sport, requiring sport-specific approaches to athlete preparation and performance enhancement.

These differences were also reflected in how athletes adapted to their new environments. Swimming integration was facilitated by pre-existing networks and residential cohesion, consistent with ecological perspectives on supportive development environments (16). In contrast, basketball integration was more status-sensitive and hierarchically structured, with athletes negotiating reduced roles and fluctuating instructional attention. However, across both sports, athletes noted the importance of peer relationships and older teammates as part of their integration into senior environments. These social dynamics were embedded within the transition process itself and functioned as an informal source of support within daily training and competition settings, particularly in basketball where institutional support services were more limited. The current findings underscore the value of horizontal, peer-based support (in contrast to top-down interventions from coaches or psychologists) in sustaining athletes through challenging periods. This emphasis on naturally occurring interpersonal support aligns with previous work on athlete development environments and the role of informal social networks in sport (9, 40), suggesting that peer interactions may represent an underutilized resource for facilitating adaptation within elite performance settings.

Although social adjustment was generally reported as manageable in both sports, access to institutional psychological support differed markedly: in swimming, such support was embedded within the system, whereas in basketball it was largely externalized, with athletes often relying on private services. This highlights how access to structured support is shaped by organizational design rather than individual need.

Beyond these integration dynamics, athletes across both sports reported broader organizational gaps, including limited structured guidance, inconsistent communication, and abrupt changes in training expectations. In swimming, these issues were particularly evident within the Academy, where high performance expectations were not always accompanied by emotional or developmental support. In basketball, athletes described a clear shift from relational coaching environments in youth sport to more transactional, performance-oriented senior systems. These discontinuities reflect the “barriers” identified in Stambulova's (5) transition model and point to broader systemic issues in talent pathway design. Overall, the findings reinforce Henriksen et al.'s (16) argument for ecologically supportive systems that integrate psychological and social continuity alongside performance demands, thereby facilitating sustained progression through elite development pathways.

A distinctive contribution of this study concerns the role of military enlistment as a structural condition shaping the junior-to-senior transition. Rather than functioning merely as an additional stressor, military service emerged as a bottleneck interacting directly with athletic timing and developmental progression. For swimmers, it represented a potential interruption of a narrow physiological peak window, whereas for basketball players it imposed daily logistical and cognitive strain within an already professionalized environment. As athletes simultaneously entered senior sport, military service, and in some cases higher education, the transition became embedded within multiple overlapping institutional commitments. Although state-sponsored arrangements provide a degree of flexibility for elite athletes, participants reported inconsistencies in implementation, with training opportunities often dependent on the discretion of military or institutional authorities. These findings extend holistic career transition models (7, 16, 23) by demonstrating how sociocultural and policy-level structures can shape athletic development trajectories, particularly in contexts where elite sport coexists with mandatory national service, with potential consequences for long-term athlete development and performance continuity.

Despite substantial structural pressures, athletes in both sports retrospectively framed the transition as formative and character-building. Notably, their reflections centered less on emotional crisis but on adaptation, maturity, and recalibrated expectations. Swimmers emphasized holistic preparation and dual-career sustainability, whereas basketball players placed greater emphasis on competitive positioning and upward mobility within professional hierarchies. These patterns suggest that the junior-to-senior transition may be experienced less as acute disruption and more as progressive recalibration within evolving performance environments, particularly when appropriate structural support and developmental continuity are maintained, allowing athletes to sustain progression toward senior-level performance.

An important consideration in interpreting these sport-specific differences is the age disparity between the two groups. The basketball players were older on average than the swimmers (M = 18.9 vs. 16.6 years), and developmental stage may therefore have contributed to some of the observed patterns. The more institutionally structured entry pathway described by swimmers may partly reflect their younger age and earlier stage of senior development, whereas the basketball players' emphasis on playing opportunities, professional positioning, and competitive accountability may be related in part to their more advanced maturity and exposure to senior competition. Similarly, differences in performance demands, social integration and support, military service, and future aspirations may have been influenced not only by the organizational characteristics of the two sports but also by the athletes' different ages and developmental stages. For example, age may have shaped social integration, as swimmers explicitly referred to the age and generational proximity of their teammates when describing their relationships. Thus, while the findings point to meaningful differences between the two sporting contexts, these differences should be interpreted with caution, as sport type and age were systematically intertwined in the present sample.

### 4.2 Practical applications

The findings of this study carry several important practical applications for elite athlete development. First, sport organizations should develop structured junior-to-senior transition programs that begin in the final stages of junior sport and continue into the early senior phase. Such programs should integrate psychological preparation with performance planning, including clear communication regarding role changes, performance expectations, and available support systems. Second, peer mentoring should be formally incorporated into transition frameworks, given its consistent importance across both sports, as it may facilitate adaptation, preserve athlete engagement, and support continuity with elite development pathways. In parallel, coach education should include explicit training on transitional sensitivity, enabling coaches to better recognize and respond to the psychological, social, and performance-related challenges associated with this developmental phase. Moving from purely results-oriented approaches toward developmentally responsive coaching may enhance athlete adaptation, long-term progression, and sustainable performance. Finally, stronger coordination should be established among clubs, federations, educational institutions, and military authorities to better manage overlapping demands experienced by young elite athletes. Clearer dual-career frameworks that integrate sporting, academic, and civic obligations may contribute to more coherent and sustainable pathways toward senior-level performance.

### 4.3 Limitation and future directions

This study has several limitations that should be acknowledged. First, it focused exclusively on male athletes in two sports, which limits generalizability of the findings. In addition, the sample size, while sufficient for thematic saturation, remains relatively small and sport-specific. Future research should therefore include female athletes to examine potential gendered differences in transition experiences, as well as extend the analysis to additional sport systems with varying levels of centralization and market orientation. The absence of perspectives from coaches and organizational stakeholders also restricts the ability to fully capture the systemic nature of the transition process.

A further limitation is the age difference between the two groups, which represents a potential confound related to the different timing of the junior-to-senior transition in the two sports. Because sport type and age were systematically intertwined in the sample, the extent to which the observed differences reflect sport-specific characteristics or developmental stage cannot be determined. Future research should consider age-matched samples or longitudinal designs to better distinguish the effects of sport type and age on transition experiences. In addition, recruitment through club coaches, together with the requirement of sufficient language proficiency to participate, may have introduced a selection bias toward more socially integrated or communicative athletes. The two subsamples also differed in progression status, as basketball players had all reached senior-level competition (Premier or National League), whereas swimmers included both Academy and club athletes. Future studies should include both progressing and non-progressing athletes in each sport to enable more balanced comparisons.

Future studies should build on these limitations in several ways. Longitudinal designs would be particularly valuable for capturing how transition experiences, identity development, and performance trajectories evolve over time. Mixed-methods approaches that combine standardized transition monitoring tools with qualitative interviews could further enhance both depth and generalizability. Research incorporating multiple stakeholders, including coaches, sport psychologists, and parents, would also provide a more comprehensive understanding of how support systems function in practice. In addition, experimental or intervention-based studies are needed to evaluate the effectiveness of targeted support strategies, such as structured mentoring programs and psychological skills workshops, in improving transition outcomes. Finally, emerging digital tools and online mentoring platforms present promising avenues for scalable and accessible support, particularly in decentralized or resource-limited sport environments.

## 5 Conclusion

The junior-to-senior transition is not a uniform developmental challenge but a sport-specific process shaped by organizational structures, performance demands, and broader institutional contexts. By comparing centralized and market-driven systems under dual institutional constraints, this study advances theoretical understanding of athletic transitions as organizationally embedded and culturally situated processes rather than solely psychological adjustments. The transition from junior to senior sport represents more than an increase in competition level; it is a multidimensional developmental passage in which identity, social integration, organizational support, and performance expectations intersect to influence progression toward senior-level performance. This study contributes to existing transition frameworks by demonstrating how sport-specific systems and institutional conditions shape athletes' developmental trajectories within elite pathways. Supporting young athletes through this transition therefore requires integrated approaches that combine performance preparation with coherent organizational structures, enabling sustainable development and long-term success in elite sport.

## Data Availability

The original contributions presented in the study are included in the article/Supplementary Material, further inquiries can be directed to the corresponding author.
